# Hot Topic Recognition of Health Rumors Based on Anti-Rumor Articles on the WeChat Official Account Platform: Topic Modeling

**DOI:** 10.2196/45019

**Published:** 2023-09-21

**Authors:** Ziyu Li, Xiaoqian Wu, Lin Xu, Ming Liu, Cheng Huang

**Affiliations:** 1 Chongqing Medical University College of Medical Informatics Chongqing China; 2 Department of Quality Management Daping Hospital Army Medical University (The Third Military Medical University) Chongqing China; 3 Department of Quality Management Xinqiao Hospital Army Medical University (The Second Military Medical University) Chongqing China

**Keywords:** topic model, health rumors, social media, WeChat official account, content analysis, public health, machine learning, Twitter, social network, misinformation, users, public health, disease, diet

## Abstract

**Background:**

Social networks have become one of the main channels for obtaining health information. However, they have also become a source of health-related misinformation, which seriously threatens the public’s physical and mental health. Governance of health-related misinformation can be implemented through topic identification of rumors on social networks. However, little attention has been paid to studying the types and routes of dissemination of health rumors on the internet, especially rumors regarding health-related information in Chinese social media.

**Objective:**

This study aims to explore the types of health-related misinformation favored by WeChat public platform users and their prevalence trends and to analyze the modeling results of the text by using the Latent Dirichlet Allocation model.

**Methods:**

We used a web crawler tool to capture health rumor–dispelling articles on WeChat rumor-dispelling public accounts. We collected information from health-debunking articles posted between January 1, 2016, and August 31, 2022. Following word segmentation of the collected text, a document topic generation model called Latent Dirichlet Allocation was used to identify and generalize the most common topics. The proportion distribution of the themes was calculated, and the negative impact of various health rumors in different periods was analyzed. Additionally, the prevalence of health rumors was analyzed by the number of health rumors generated at each time point.

**Results:**

We collected 9366 rumor-refuting articles from January 1, 2016, to August 31, 2022, from WeChat official accounts. Through topic modeling, we divided the health rumors into 8 topics, that is, rumors on prevention and treatment of infectious diseases (1284/9366, 13.71%), disease therapy and its effects (1037/9366, 11.07%), food safety (1243/9366, 13.27%), cancer and its causes (946/9366, 10.10%), regimen and disease (1540/9366, 16.44%), transmission (914/9366, 9.76%), healthy diet (1068/9366, 11.40%), and nutrition and health (1334/9366, 14.24%). Furthermore, we summarized the 8 topics under 4 themes, that is, public health, disease, diet and health, and spread of rumors.

**Conclusions:**

Our study shows that topic modeling can provide analysis and insights into health rumor governance. The rumor development trends showed that most rumors were on public health, disease, and diet and health problems. Governments still need to implement relevant and comprehensive rumor management strategies based on the rumors prevalent in their countries and formulate appropriate policies. Apart from regulating the content disseminated on social media platforms, the national quality of health education should also be improved. Governance of social networks should be clearly implemented, as these rapidly developed platforms come with privacy issues. Both disseminators and receivers of information should ensure a realistic attitude and disseminate health information correctly. In addition, we recommend that sentiment analysis–related studies be conducted to verify the impact of health rumor–related topics.

## Introduction

### Background

Health rumors were first proposed as a significant public health issue in 2005 [1]. Over the past decade, health rumors have attracted much attention in China. Currently, health rumors have become an unavoidable social problem, causing trouble to netizens [2]. Health rumors are presented as risk information with unsubstantiated facts that may cause individuals or groups to engage in inappropriate or unreasonable behavior related to their health [3]. The rampant spread of health rumors has given rise to a new phenomenon called the information epidemic [4]. An information epidemic is an excess of information, which could be both correct and incorrect, thereby making reliable sources of information elusive; in severe cases, it may even endanger people’s health [5,6].

### Harms of Health Rumors

The crisis of public opinion social networks, which are characterized by frequent rumors, is often accompanied by public health emergencies [7]. As a significant medium for information creation, release, and dissemination, social media platforms have become the new approach for individuals to obtain health information [8,9]. A survey [10] has found that 63.26% of the users would use social media to search for health information. Moreover, a study on users’ preferences for health information channels showed that users are more willing to use social media to search for health information rather than online health information platforms [11]. Although social media has become the most important channel for users to obtain health information, it is also filled with several useless and even harmful health-related misinformation, including uneven quality of information, serious information pollution, and difficulty distinguishing between true and false information [12]. Health rumors that spread widely on social media may affect public health behaviors and even be threatening to their lives if treatment is delayed due to the rumors. Since the outbreak of COVID-19, not only did the disease rapidly spread worldwide but also the output and impact of rumor information increased significantly. The World Health Organization carried out a series of communication campaigns to combat the spread of misinformation on the spread of the COVID-19 disease and the information epidemic in 2021 [13-15]. In particular, among rumors generated by public health crises, the proportion of health rumors is higher and the social impact is more serious [16]. In China, epidemic-related rumors in 2020, including “COVID-19 is a biological weapon” and “Shuang-Huang oral liquid can treat COVID-19,” consecutively emerged on social media platforms, including WeChat and Weibo, and subsequently filled the cyberspace, causing panic to the public [17].

### Social Media Platforms in China

Social media platforms have an impact on how one manages and confirms or busts health rumors [18]. Research on health rumors on social media in the United States and India are mainly categorized as epidemic-related health information, information epidemic, and anti-vaccine and vaccine hesitancy. However, research on the health information that is widely circulated on the social media is lacking [19,20]. Some studies have mainly focused on Twitter, YouTube, Facebook, and other social media platforms [21]. Studies on health rumors [22] in China have mainly focused on Weibo articles without any research on WeChat articles. However, there are large differences in the spread of rumors across different platforms. For example, there is a big difference between WeChat and Weibo regarding the interpersonal relationships of the users of these platforms. WeChat is a “strong relationship” network of relatives, friends, colleagues, and acquaintances, whereas Weibo is a “social relationship” network composed of people with common hobbies and interests. The information acquisition and the interpersonal communication in WeChat have a relatively strong direct effect on the acquisition of online and offline social support in WeChat [23]. Compared with rumors in Weibo, those in WeChat are more easily accepted by readers. Further, the closed and private nature of WeChat makes it more difficult to clarify rumors, and the role of rumors is more persistent in WeChat. Therefore, to explore the prevalence of health rumors according to the typical events in each period, we decided to consider WeChat as the research platform. The results of our study can effectively prevent the subsequent information epidemic of health rumors and provide evidence-based recommendations for information exchange and health communication.

## Methods

### Data Collection

In China, WeChat is the most popular social media platform, and WeChat official accounts provide high-quality health-related articles [24]. It allows users to search keywords to see related accounts and articles [25]. Using this function, we found dozens of WeChat rumor-dispelling public accounts such as “internet rumor–dispelling platform,” “rumor-dispelling platform,” and “Shanghai network rumor dispelling,” by searching with keywords such as “rumor-dispelling,” “health rumor–dispelling,” “rumor,” and “health rumors.” These accounts are created by Chinese city governments, and the articles they refute are representative of those that disrupt social order and cause serious public health concerns. To determine whether the trend of rumor prevalence changed over time, we used the Python web crawler to collect health rumor–related dispelling articles between January 1, 2016, and August 31, 2022. After extracting the data, we formed a data set that contained the time of posting, the content of each article, and the name of the author who posted the article. We collected 9410 articles on related official WeChat accounts. Prior to the analysis, we cleaned up the data after completing the preprocessing steps. The data cleanup consisted of removing redundant data, including advertisements and data sets irrelevant to health rumors. Duplicate data were deleted. Images were removed, as image texts could not be extracted. Additionally, commonly used Chinese pause words such as “a,” “of,” and “it” were removed [26,27]. The document-term matrix was constructed, and term frequency-inverse document frequency was used to process the data. After cleaning and noise reduction, 9366 valid data were obtained [28].

### Data Analysis

We compared several models and found that the nonnegative matrix factorization model is a matrix decomposition technique that tries to decompose a nonnegative matrix into a product of 2 nonnegative matrices, where these 2 matrices represent the features and feature combination weights of the data [29]. Therefore, nonnegative matrix factorization is more suitable for matrix decomposition of nonnegative data and is applicable to data such as images, audio, and gene expression [30,31]. In addition, Latent Dirichlet Allocation (LDA) is a probabilistic-based generative model that better reflects the stochastic relationship between documents and topics and helps to capture the underlying structure of textual data [32]. As an unsupervised machine learning method, the LDA topic model uses Dirichlet distribution for probabilistic modeling at 3 levels, namely, document, topic, and word [33]. It performs semantic similarity computation on topics, documents, and keywords. By providing text input and by setting the desired number of topics, LDA automatically generates a set of topics, assigns words to topics, and assigns a topic ratio to each document. The LDA topic model is effective in transforming text categorization problems into machine-understandable linguistic problems with improved accuracy [34,35]. By using Gibbs sampling—a method for estimating the marginal distributions of the variables of interest—the LDA topic model can determine the topics in the data pool. Therefore, LDA is more suitable for processing textual data, and topic modeling of text is used to discover potential topics in textual data. Finally, the hierarchical Dirichlet process model is an extension of the LDA model that introduces a hierarchical structure. In the hierarchical Dirichlet process model, it is assumed that each document consists of an infinite number of topics, where each topic is sampled by the upper and lower Dirichlet processes, and a hierarchical topic structure can be learned [36]. However, due to the more complex structure of the hierarchical Dirichlet process model, more parameters need to be learned. This also increases the model error and instability [37]. Overall, LDA as a classical model and the training method is more mature and stable. This study needs a stable and repeatable result of topic modeling; therefore, LDA would be a better choice. Several previous studies [38,39] have demonstrated the effectiveness of the model in research topic mining and research trend prediction.

**Figure 1:**
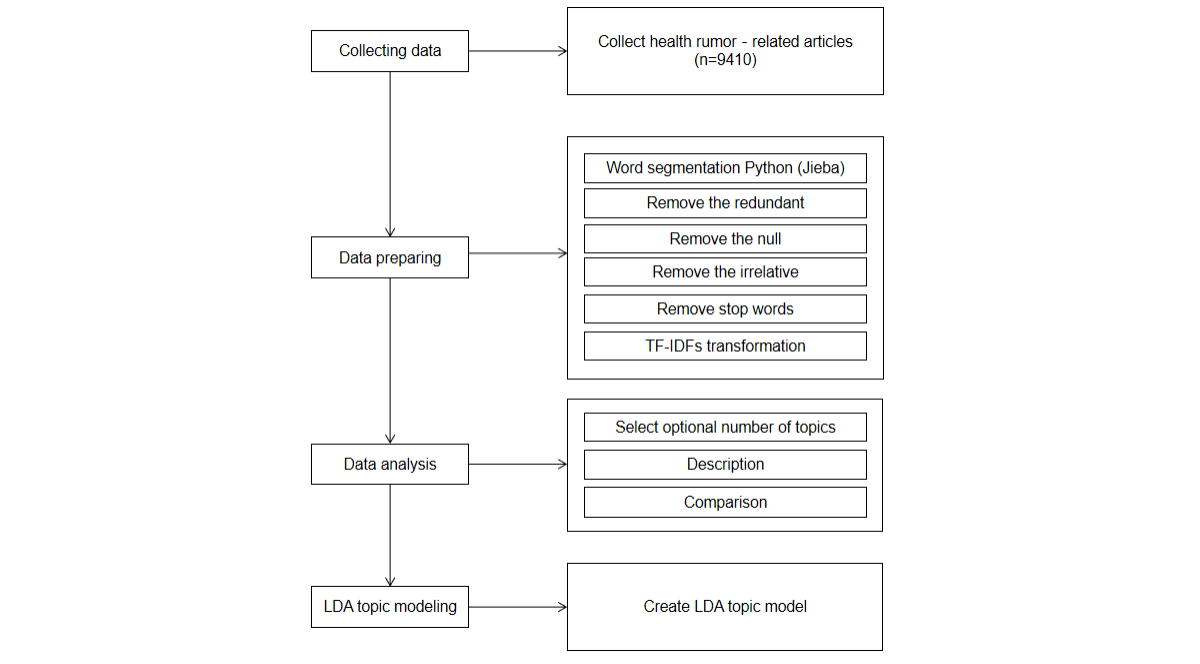
Flowchart in this study. LDA: Latent Dirichlet Allocation; TF-IDF: term frequency–inverse document frequency.

Before applying LDA topic modeling, data were first processed (Figure 1). Python 3.9.7 was used to clean the data to avoid the noise that affects the topic modeling. Then, Jieba word segmentation was used to segment the data. We used several common Chinese deactivation word lists such as the Baidu deactivation word list, which was used as the basic word list, and we added high-frequency but nonactual words to the deactivation word list according to this study. A deactivation word list was developed for this study when the data were eliminated and the data were integrated. Domain-specific research requires the construction of relevant domain lexicons to improve the effect of word segmentation. Subsequently, the separated keywords are extracted using the k-means algorithm, and the data are clustered to form K clusters corresponding to the optimal number of topics. This step can greatly improve the performance of the algorithm and stabilize the random inference of the potential Dirichlet allocation [40].

**Figure 2:**
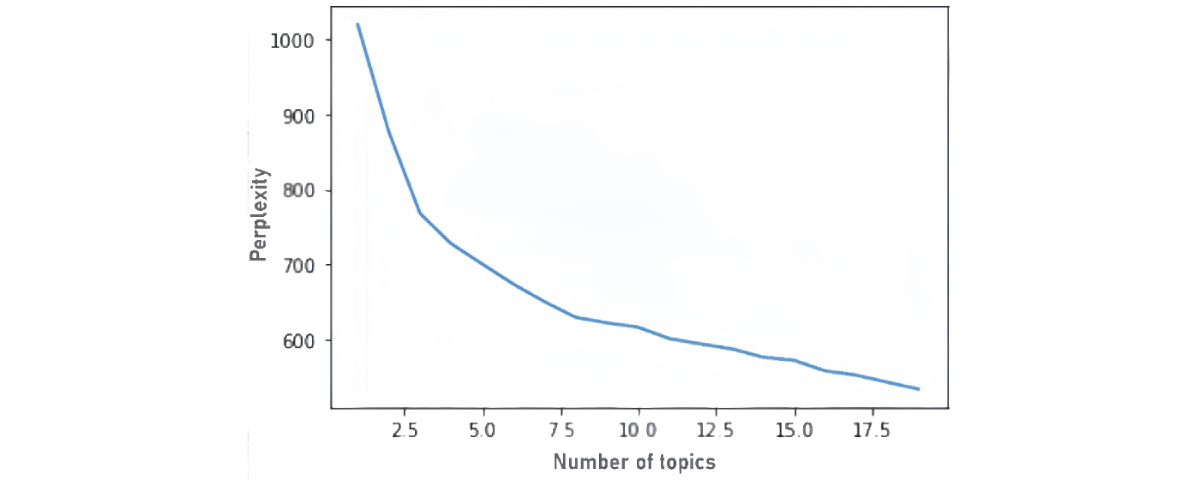
Topic perplexity score curve for evaluating the performance of the topic model.

The number of topics is an important parameter of LDA that needs to be set manually. In order to determine the appropriate number of LDA topics, this study employs sensitivity analysis by using perplexity as a judgment method, which has been proposed for selecting the appropriate number of LDA topics [41]. Perplexity is a common method for determining the number of topics, with smaller perplexity values representing a better model fit. As shown in Figure 2, the perplexity decreases as the number of topics increases. However, it is often difficult to interpret the selection of the number of themes by relying only on statistical methods. Therefore, we further evaluated the model effect by sensitivity analysis, which can help us better adjust the LDA model and obtain an interpretable and stable topic model [42]. We constantly changed the number of themes, K, by fixing other parameters and by observing the changes in the model results. When K is less than 8, the clarity of themes decreases; when K is greater than 8, the themes are semantically repetitive. Therefore, we found that when the number of themes is 8, the consistency score is the highest and the model is the most stable. Therefore, we chose 8 as the number of themes. To elaborate the themes, we also generated the theme names based on the given keywords.

### Ethics Approval

This study was approved by the ethics committee of Chongqing Medical University on September 15, 2020.

## Results

### Topic Modeling

**Figure 3:**
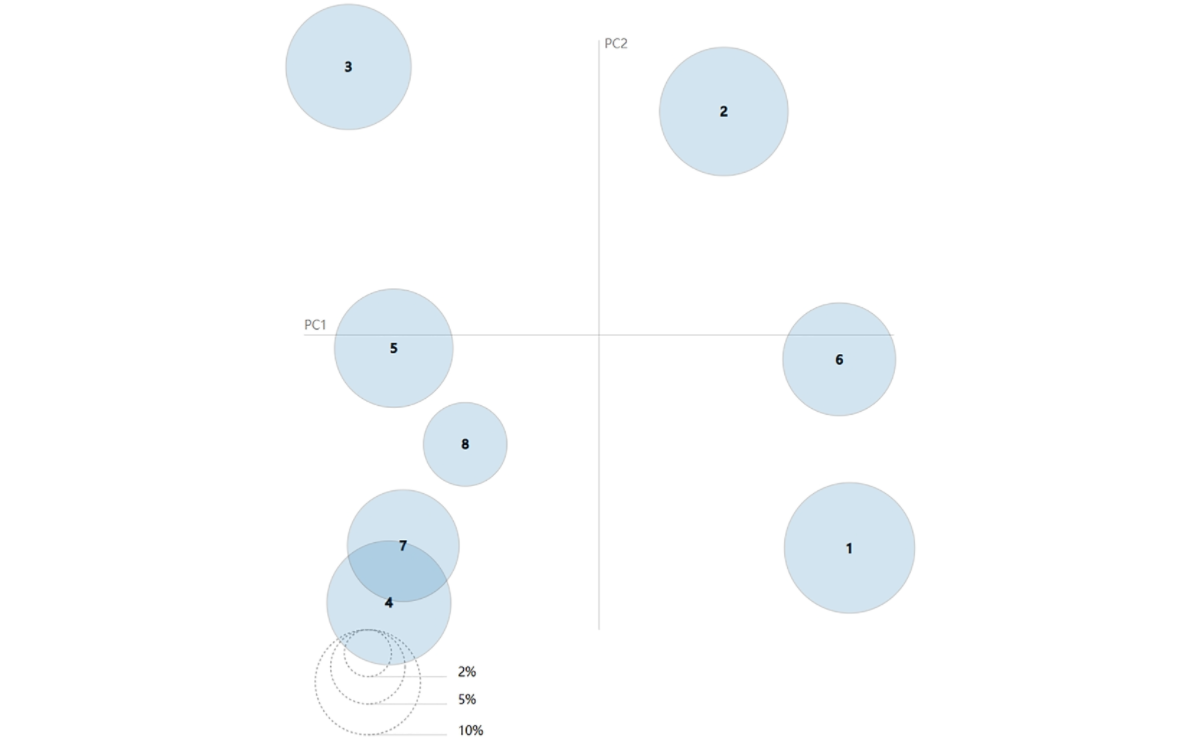
Intertopic distance map. The size of the circles indicates proportionally the number of words that belong to each topic. Th 8 topics are (1) prevention and treatment of infectious diseases, (2) disease therapy and its effects, (3) food safety, (4) cancer and its causes, (5) regimen and disease, (6) rumors of transmission, (7) healthy diet, and (8) nutrition and health. PC: principal component.

By applying LDA topic modeling, we generated 8 groups of topics and obtained the top 10 keywords with the frequency of each group of topics. Each topic content was generated based on its associated set of keywords. However, no matter how advanced the statistical measurements are, the complexity of the language makes it impossible to ensure that the output can be interpreted in words. Therefore, we added human interpretation to analyze the topic. Moreover, topics were named according to the corresponding keywords to illustrate the topic. Based on the results obtained and the keywords for each topic, we were able to classify the rumors into the following 8 topics: (1) prevention and treatment of infectious diseases, (2) disease therapy and its effects, (3) food safety, (4) cancer and its causes, (5) regimen and disease, (6) rumors of transmission, (7) healthy diet, and (8) nutrition and health. Figure 3 shows the intertopic distance map, where each topic is represented by a circle. The popularity of each topic is indicated by the area of the circle. The center of each topic was determined by calculating the distance between the topics. We used multidimensional scaling to represent intersubject distances on a 2D plane [43]. As there was overlapping of topics, the topics were further divided into the following 4 themes: public health, disease, diet and health, and spread of rumors. Simultaneously, we determined the number and proportion of words that belonged to each topic by counting the number of articles under each topic to understand the specific interest in each topic. For example, the ratio for each topic is the number of debunked articles on that topic divided by the total number of debunked articles (N=9366). Finally, we categorized the topic name and keywords as well as the statistical results as shown in Table 1.

**Table 1:** Topics and keywords formulated by Latent Dirichlet Allocation topic modelling (N=9366).

Theme, topic			Keywords		Values, n (%)
**Theme 1: Public health rumors**					
	Topic 1: Prevention and treatment of infectious diseases	Virus, vaccine, COVID-19, pneumonia, mask, spread, detect, case, and influenza		1284 (13.71)	
**Theme 2: Disease rumors**					
	Topic 2: Disease therapy and its effects	Patient, drug, symptom, disease, child, children, doctor, taking, hypertension, and blood vessel		1037 (11.07)	
	Topic 4: Cancer and its causes	Research, cancer, patient, risk, tumor, cell, experiment, gene, factor, and disease		946 (10.10)	
	Topic 5: Regimen and disease	Skin, exercise, body, effect, child, human body, time, female, muscle, and disease		1540 (16.44)	
**Theme 3: Diet and health rumors**					
	Topic 3: Food safety	Food, product, human body, content, additive, ingredient, standard, food, substance, and formaldehyde		1243 (13.27)	
	Topic 7: Healthy diet	Edible, fruit, poisoning, food, vegetable, plant, bacteria, banana, growth, and hormone		1068 (11.40)	
	Topic 8: Nutrition and health	Food, nutrition, fat, diet, protein, content, milk, food, egg, and human body		1334 (14.24)	
**Theme 4: Spread of rumors**					
	Topic 6: Rumors of transmission	Network, information, news, network transmission, spread, netizen, spread, expert, official, and media		914 (9.76)	

### Topic Analysis of Health Rumors

As shown in Table 1, statistics related to the prevalence of health rumors can provide additional insight into the subsequent analysis of the prevalence of each rumor topic. The topics of the rumors have been explained below as per the order of their frequency in the articles.

#### Topic 5

According to the research results, we summarized regimen and disease in topic 5, and the keywords were skin, exercise, body, human body, muscle, and disease. We found rumors, including “drinking can make the body warmer” and “doing yoga is harmful to the body.” In all articles, topic 5 ranked first, accounting for 16.44% (1540/9366) of all the articles.

#### Topic 8

The keywords in topic 8 focused on food, nutrition, fat, diet, protein, and milk. Therefore, we summarized topic 8 as “nutrition and health.” We found rumors, including “drinking bone soup can provide calcium” and “drinking vinegar can soften blood vessels.” Debunking articles under the “nutrition and health” theme ranked second, comprising 14.24% (1334/9366) of all the articles.

#### Topic 1

Infectious diseases are a hot topic of social concern. Particularly, since the outbreak of COVID-19 in late 2019, several infectious disease–related health rumors have been generated. Through topic modeling, we obtained keywords, including virus, vaccine, COVID-19, epidemic, pneumonia, spread, and influenza. Consequently, we summarized topic 1 as “the prevention and treatment of infectious diseases.” Among the articles we collected, we found health rumors such as “COVID-19 stays in the throat for 4 days, and gargling can eliminate the virus” and “drinking more tea can strengthen immunity and prevent the novel coronavirus.” Rumor articles related to topic 1 ranked third, accounting for 13.71% (1284/9366) of all the articles.

#### Topic 3

According to our research results, we found that the number of rumor articles in topic 3 was 1243, accounting for 13.27% of all the articles. Among them, the keywords of this topic were focused on food, product, content, additive, ingredient, standard, food, and hazard. By sorting through these keywords, we defined topic 3 as “food safety.” Some of the articles were similar to those that read “eating watermelon and peaches at the same time will lead to poisoning” and “eating too much honey will cause premature puberty.” Topic 3 ranked fourth based on the number of articles.

#### Topic 7

From the results of topic modeling, topic 7 was composed of keywords, including edible, fruit, poisoning, food, bacteria, hormones, and toxins. Therefore, we summarized topic 7 as “healthy diet.” From the collected articles, we extracted health rumors, including “yolks are high in cholesterol, so eating too much is bad for your health” and “cherries can cause cyanide poisoning if you eat too much.” According to the statistical results, topic 7 ranked fifth, accounting for 11.40% (1068/9366) of all the articles.

#### Topic 2

Based on keywords, including patient, drug, symptom, disease, children, hypertension, blood vessel, and hospital, we summarized topic 2 as “disease therapies and effects.” Additionally, from the collected articles, we extracted health rumors such as “wearing blue light glasses can prevent myopia” and “folk prescription can be used to treat hypertension.” According to the statistical results, topic 2 ranked sixth, accounting for 11.07% (1037/9366) of all the articles.

#### Topic 4

Through the research results, we found that the keywords of topic 4 mainly focused on research, cancer, risk, tumor, cell, experiment, gene, alcohol, anticancer, and drugs. Therefore, we summarized topic 4 as “cancer and its causes.” Furthermore, from the articles we collected, we found rumors such as “drinking more liquor can fight cancer” and “if the body constitution is acidic, it is more prone to cancer.” This topic ranked seventh, accounting for 10.10% (946/9366) of the total number of articles.

#### Topic 6

With the rapid development of mobile health, the internet has increasingly become a convenient channel for obtaining health information. Concurrently, it also provides a source for health rumors to arise and spread. Based on the results of keywords, including network, news, spread, public, netizen, experts, official, and media, we summarized topic 6 as “rumors of transmission.” Topic 6 ranked eighth, accounting for 9.76% (914/9366) of the total number of articles.

## Discussion

### Principal Findings

**Figure 4:**
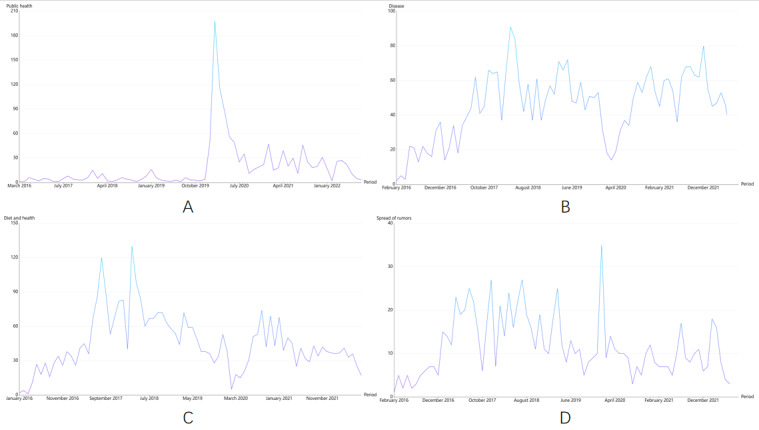
Health rumor trend chart of each type of rumor from January 1, 2016, to August 31, 2022. A. Public health rumors. B. Disease rumors. C. Diet and health rumors. D. Spread of rumors.

We analyzed 9366 health rumor–related articles from January 1, 2016, to August 31, 2022, collected from WeChat official accounts. The findings of our study can be useful for future studies on health rumor trend monitoring and characteristic analysis. Moreover, our study provides a scientific approach for the management of spread of health rumors on social media platforms and can effectively prevent the subsequent information epidemic of health rumors and provide evidence-based recommendations for information exchange and health communication. As per the results of topic modeling in our study, we divided the 8 topics of health rumors into 4 themes, of which 3 themes were based on the types of rumors and 1 theme was based on the spread of rumors. The line chart of the development trend of the 3 types of rumors is shown in Figure 4. The development trend of the 3 types of rumors showed that the number of rumors related to theme 1 (public health) tended to be flat in the early stage; however, at the end of 2019, the number of rumors reached the peak in a short time owing to the sudden outbreak of COVID-19, which was a public health emergency. Initially, the source of COVID-19 was unclear, therefore, the rumors of its origin or transmission or causes could not be accurately verified with evidence-based proofs. Consequently, the number of COVID-19–related rumors rapidly increased in 2020. However, before the old rumors were resolved, new rumors consecutively emerged, and the old and new rumors became intertwined, thereby making the number of rumors reach its peak. When the epidemic situation came under effective control, China vigorously promoted COVID-19 vaccinations and established equitable herd immunity. China’s effective prevention and control measures for the epidemic greatly reduced the public’s fear and anxiety, thereby gradually reducing the number of rumors [44]. Our findings suggest that WeChat official accounts can post health rumor–related disinformation articles on COVID-19, virus infection, and vaccine intervention. Some of the rumors regarding epidemic prevention and control were posted by official government agencies, which made the public angry, leading to the decline of the government’s prestige and lack of public confidence, thereby creating a negative impact on the social fight against the epidemic. Such rumors could even lead to a nationwide crisis of social trust, which would negatively impact the government’s public health function. Thus, public health–related rumors can seriously harm WeChat users and the society [45].

We found that the number of rumors related to theme 2 (disease) continued increasing since the data collection in 2016, and it remained at a high and stable level. This trend did not change until the emergence of the COVID-19 pandemic. In early 2020, as the pandemic spread and rumors circulated, the public diverted their attention to fears of contracting the new disease and ways to prevent the virus. Therefore, Figure 4 shows that the number of rumors related to theme 2 continued to decline from December 2019 to March 2020, reaching the lowest value. However, although the COVID-19 outbreak has been effectively controlled over time, rumors related to theme 2, including “new coronavirus causes severe infections and its complications” and “new coronavirus pneumonia can cause a decline in immunity,” have re-emerged in the society; therefore, the trend of these rumors bounced back from June 2020 to February 2022. We observed that among the rumors of theme 2, topic 5 (regimen and disease) had the highest proportion of rumors because in different periods, Chinese individuals have frequently paid more attention to regimens, for example, Chinese people have always attached great importance to traditional Chinese medicine such as acupuncture and fire-cupping not only because it can cure diseases but also because it can nourish the body. Even before the COVID-19 pandemic, there were some health-related rumors without any scientific basis, including “shampooing with beer will protect your hair” and “exercising on an empty stomach will help you lose weight” [46,47]. However, after the COVID-19 outbreak, the health rumors that appeared were “taking hot showers can prevent the novel coronavirus” and “basking in the sun can eliminate the novel coronavirus,” resulting in a greater number of rumors related to topic 5 than the number of rumors related to other topics. The popularity trend of the rumors related to theme 2 shows that health information related to diseases has always been a hotspot of people’s attention; therefore, the popularity of disease rumors remains stable for a long time. The results of topic modeling also showed that disease rumors are mainly categorized into 3 topics, which are closely related to people’s health: disease therapy and its effects, cancer and its causes, and regimen and disease. The analysis of these 3 topics showed that disease rumors harm people in many ways. Disease rumors not only seriously hinder patients from obtaining correct and timely diagnosis and treatment information but also reduce the efficacy of patients’ treatment. Therefore, our study highlights that the public should not only improve their personal health literacy and learn to identify health rumors but be able to differentiate accurate information from misleading ones to prevent deterioration of their diseases by believing rumors. In addition, the government should pay more attention to health rumors to prevent the public’s health from being threatened by the rampant spread of disease rumors.

A healthy diet is a key topic of concern in every country. Therefore, the number of rumors related to specific diets is generally high. The trend of theme 3 (diet and health) indicates 2 peaks in a year (Figure 4), particularly in August 2017 and March 2018. The first peak occurs in August. Since high temperatures in summer can lead to food spoilage, summer is the peak period for food safety incidents, which also suggests that health rumors are easy to spread during this period. The second peak occurs in March after the traditional spring festival in China. Not only is this the time for people to have large meals but also the time for various kinds of overeating and frequent food poisonings, leading to a continuous increase in the number of health rumors. In 2020, the popularity of theme 3 decreased to its lowest level due to the COVID-19 pandemic. For a while, rumor mongers turned all their attention to COVID-19. However, by observing the subsequent rumor articles, we found that there were rumors on the internet, including “COVID-19 intervention by improving healthy diets and health products,” which caused the trend of theme 3 to rebound in July 2020. From the results of theme 3, we can draw the following conclusion: it is urgent to dispel rumors on healthy diets because a healthy diet not only concerns people’s physical condition but also represents the stability of the country and society. Therefore, health-related rumors on the internet should be regulated and controlled because rumors of specific diets can have a serious impact on the society [48].

We analyzed not only the prevalence trend of rumors but also the characteristics of rumor propagation. In this study, by observing the content characteristics of the collected articles, we found that the rumor articles collected earlier mostly appeared in the form of text and had a single content form. However, in the later stage, the collected rumors were mostly circulated in the form of text content combined with pictures or videos, thereby increasing the rumors’ credibility. WeChat is one of the most popular messaging tools used for communication. Numerous WeChat groups and public accounts have built a broad platform for health communication. However, along with the development of WeChat, many issues and hidden dangers have emerged with this platform. For example, WeChat is a social media platform with a large number of users, but the information shared on this platform is relatively closed and not transparent [49,50]. Consequently, WeChat became a disastrous platform for the spread of health rumors.

### Key Learnings

Our findings could facilitate the prevention and control of health rumors in the future, in terms of both the areas in which health rumors occur and the ways in which they are spread. First, as health rumors occur more often in the medical field, these messages aim to popularize health science and improve health literacy among readers, but people interpret these messages out of context and exaggerate them, thereby distorting the audience’s understanding of health information. Therefore, the governance of health rumors requires not only the WeChat platform but also the joint efforts of relevant governments and medical institutions to verify and disseminate health information after vetting. Second, sharing information anonymously on mobile and web-based platforms is on the rise. Many users spread rumors on social media under anonymous usernames without any connections to online personal identities [51,52]. Therefore, it is recommended to implement real-name authentication for health information senders on the WeChat platform to ensure the authenticity and traceability of information. This can ensure that health information is more reliable in all aspects. Third, the use of artificial intelligence in the WeChat platform for information review can help in viewing huge amount of health information in a timely and effective manner every day. The automatic screening of big data and artificial intelligence, supplemented by manual auditing, has improved the efficiency of health information auditing on WeChat platforms. Finally, WeChat should strengthen the fact-checking of articles published on public accounts. Fact review can effectively ensure the objectivity of the articles; they not only improve the credibility of official accounts but also ensure the high quality of the published articles. Before each article is published, fact-checking can not only confirm the accuracy of the content but also ensure the authenticity of the content. On the one hand, it can avoid readers’ misunderstanding of the content of the article, while on the other hand, it can reduce the wanton spread of health-related misinformation on the network. It will truly stop rumors at the source and filter the content posted in the network environment. Furthermore, the public accounts in this study are mostly government departments, medical institutions, and officially verified rumor-refuting accounts [51]. We find that the scope and influence of rumor spread in chat tools are small, and most rumors are clarified by the government authorities. In contrast, rumors circulating on Weibo have a greater impact and will attract the attention of higher level departments, which will then intervene. These findings highlight the significance of coordinating the role of central and local agencies in establishing mechanisms to refute rumors, improve feedback mechanisms, and maximize the self-filtering capacity of social media.

### Limitations

This study has several limitations. First, content analysis was performed on articles generated by WeChat official accounts. Because the rumor that was initially circulated would be removed after refutation, obtaining the original rumor articles was impossible. Second, this study only analyzes the content of the articles. Therefore, in future studies, the number of likes for the articles and the comments of the users should be collected, which can be used for emotional analysis experiments. Lastly, not all rumors have been resolved. In contrast, there remain several rumors in the network that need to be proved by scientific experiments to reach a conclusion. Therefore, unconfirmed rumors should be analyzed in future studies.

### Conclusion

The emergence of health rumors hinders the spread of accurate health information and damages the environment of health information release. This situation has also affected the credibility of healthy communication on social media and created a crisis of confidence. Moreover, the spread of health rumors has aggravated public panic, bringing shock and trouble to social stability. We collected discredited articles on health rumors from WeChat official accounts and used topic modeling to analyze the content of the articles. This approach could help the society better understand the negative effects of health rumors spread through social media. We believe that governments need to strengthen the control of the spread of health rumors on different platforms, increase the punishment for the spread of health rumors, and vigorously publicize the harm caused by health rumors. Owing to the uneven cultural level of the internet users, they lack recognition of the authenticity of information. Therefore, they may become rumor victims and may potentially become rumor spreaders. The governance of health rumors is a slow process, and it takes time to test the results of its management. We should deal with the problems that social networks bring in the process of rapid development and clarify the idea of governance. Both disseminators and receivers of information should ensure a realistic attitude and disseminate health information correctly. We suggest that social media platforms develop more rumor-refuting applications or WeChat mini-programs. Furthermore, the government should encourage the creation of more rumor-dispelling public accounts to eradicate rumors.

## Abbreviations

LDA: Latent Dirichlet Allocation

## Data Availability

The data sets generated during and analyzed during this study are available from the corresponding author on reasonable request.
